# First Report of a Hollow Cranial Crest in an Early-Diverging Duck-Billed Dinosaur, with Implications for Convergent Evolution of Acoustic Signaling

**DOI:** 10.3390/biology15080615

**Published:** 2026-04-13

**Authors:** Qingyu Ma, Yubo Ma, Chao Tan, Jian Chen, Yu Lin, Ming Xiao, Hui Dai, Guangbiao Wei, Jordan C. Mallon, Jun Wang, Han Yao, Zhengting Zou, Hai Xing

**Affiliations:** 1 Chongqing Institute of Paleontology, Chongqing 401122, China; ma.qingyu@foxmail.com (Q.M.); mayubo.cip@foxmail.com (Y.M.); tan-c@foxmail.com (C.T.); chenjian.cip@foxmail.com (J.C.); bestrainner@foxmail.com (Y.L.); xiaomingshuai@foxmail.com (M.X.); dhui6251@163.com (H.D.); elephantfossil@aliyun.com (G.W.); 2 Chongqing Bureau of Geological and Mineral Resource Exploration and Development, Chongqing 401121, China; 3 Chongqing Key Laboratory of Paleontology and Paleoenvironment Co-Evolution (Sichuan-Chongqing Joint Construction), Chongqing 401122, China; 4 Beaty Centre for Species Discovery and Palaeobiology Section, Canadian Museum of Nature, Ottawa, ON K1P 6P4, Canada; jmallon@nature.ca; 5 Department of Earth Sciences, Carleton University, Ottawa, ON K1S 5B6, Canada; 6 School of Earth Sciences, Yunnan University, Kunming 650500, China; junwang@ynu.edu.cn; 7 School of Earth and Planetary Sciences, China University of Geosciences, Wuhan 430074, China; hyao51247@gmail.com; 8 State Key Laboratory of Animal Biodiversity Conservation and Integrated Pest Management, Institute of Zoology, Chinese Academy of Sciences, Beijing 100101, China; zouzhengting@ioz.ac.cn; 9 Department of Geosciences, National Natural History Museum of China, Beijing 100050, China

**Keywords:** *Qianjiangsaurus changshengi*, accessory endonasal cavities, hollow supracranial crest, low-frequency vocalizations, convergent evolution

## Abstract

Cranial crests have evolved multiple times in the evolutionary history of vertebrates, serving primarily for visual display. In duck-billed lambeosaurines, one of the most successful dinosaur clades of the Late Cretaceous, the cranial crest became hollow along the paired premaxillae and nasals, and was secondarily selected as a resonating structure atop the skull roof, facilitating acoustic signaling. Here we report the first instance of a hollow supracranial crest in a non-lambeosaurine ornithopod dinosaur, the early-branching hadrosauroid *Qianjiangsaurus changshengi*, where the paired accessory endonasal cavities just above the nasal cavity proper occur following the dorsoventral thickening of the nasals. This novel nasal cavity configuration is associated with the helmet-like hollow supracranial crest solely formed by the nasals. Comparative resonance modeling suggests that the nasal cavity of *Q. changshengi* could amplify low-frequency vocalizations similar to those of late-branching lambeosaurines. Seven analogous skull features (including the hollow supracranial crest) and similar low-frequency acoustic capabilities of nasal cavities between *Q. changshengi* and late-branching lambeosaurines reveal a striking morphological and functional convergence that would likely facilitate safer, more efficient social communication among hadrosauroids. This convergence can be explained by adaptive evolution under similar selection pressures, combined with developmental constraints due to gene pleiotropy.

## Simple Summary

Convergent evolution is a peculiar biological process in which distinct taxa or lineages independently evolve analogous features, structures and functions, to adapt to similar necessities. Here we present a classic instance of morphological and functional convergence of the hollow cranial crest among hadrosauroid dinosaurs, based on an excellently preserved skull of the early-branching hadrosauroid *Qianjiangsaurus changshengi* newly recovered from southwest China, as well as comparative resonant frequency evaluations of its unique endonasal (‘within the nasal bone’) cavities using CT scans and mathematic calculations. The overgrown nasal crest with a novel internal structure in *Q. changshengi* is morphologically comparable to but structurally non-homologous with the greatly developed hollow supracranial ornamentation and elongate nasal passages seen in lambeosaurines, and thus hugely changes our notions on the cranial evolution of late-branching ornithopod dinosaurs.

## 1. Introduction

Cranial crests have independently emerged multiple times in the evolutionary history of vertebrates and primarily function as visual display in some extant squamates and birds (e.g., chameleons and cassowaries) [1,2], and likely in some extinct pterosaurs, theropods (e.g., *Monolophosaurus*, *Spinosaurus*, *Guanlong* and some oviraptorosaurians) and brontotheres [3,4,5]. Among duck-billed dinosaurs (Hadrosauroidea) and their closest relatives, a hollow supracranial crest has long been regarded as unique to Lambeosaurinae, a major late-branching clade of the Late Cretaceous northern hemisphere [6,7,8,9,10,11]. This elaborate bony structure is basically formed by the overgrown paired premaxillae and nasals atop the skull roof, and is typically helmet-like or tubular in lateral profile, seen in such examples as *Hypacrosaurus*, *Parasaurolophus* and *Olorotitan* [12,13,14,15]. The crest is hollowed by a greatly hypertrophied nasal cavity convoluted and variable in shape taxonomically and ontogenetically, including a pair of elongate premaxillary passages homologous with the nasal vestibule, an enlarged, elevated common median chamber where the passages converge, and paired, slightly winding lateral diverticula that straddle and communicate with the median chamber [8,16]. The development of the nasal cavity in lambeosaurines suggests a potential vocalization function via acoustic resonance, perhaps for intraspecific communication, in addition to visual display, rather than physiological functions like olfaction detection and thermoregulation [8,9,10,16,17,18,19,20,21]. By contrast, all known non-lambeosaurine ornithopods possess either a flat-headed skull or a solid supracranial crest mainly formed by the nasals [10,22,23,24]. The hollow supracranial crest is thus considered a novel bony configuration vital for the survival and social interaction of lambeosaurine dinosaurs via low-frequency vocalizations, and represents a significant evolutionary adaptation enabling gregariousness in this group [9,16,20,25].

*Qianjiangsaurus changshengi*, the second formally named hadrosauroid dinosaur from south China, is previously known only from the holotype, a partial skeleton including only the predentary and left dentary from the skull [26]. During the summer of 2024, the Chongqing Municipal Bureau of Planning and Natural Resources organized a paleontological excavation at a new locality of the Zhengyang area (GPS coordinates: 29°27′31″ N, 108°48′15″ E), ~1.5 km north of the holotype quarry of *Q. changshengi* in Qianjiang District of southeast Chongqing Municipality (Supplementary Materials, Figures S1 and S2). This excavation yielded a well-preserved hadrosauroid skull (CIP V0002) missing small parts of the facial skeleton and lower jaw. The skull came from the top of the Upper Cretaceous Zhengyang Formation that is stratigraphically comparable to the horizon of the *Q. changshengi* holotype. The dentary of the newly collected skull shares a series of salient features with that of the *Q. changshengi* holotype, such as the nearly vertical coronoid process bearing a strongly anteroposteriorly expanded apex; a shortened, almost horizontal edentulous margin posterior to the symphysial process; a moderately ventrally deflected anterior dentary ramus; a maximum of five teeth per alveolus (including three functional ones); each tooth crown lingually ornamented with a slightly distally offset primary ridge, a short secondary ridge mesially and a slender faint ridge distally; and mid-dentary tooth crowns with an average height/width ratio of ~2.5. Considering the perfectly overlapping stratigraphic and osteological data between the newly collected skull and the *Q. changshengi* holotype, we confidently ascribe this skull to that taxon. The new skull is not only an important supplement to the cranial anatomy of *Q. changshengi*, but also exhibits a novel internal morphology of the nasal cavity associated with a bizarre hollow nasal crest anterodorsal to the skull roof. This significant discovery reveals a previously hidden morphological diversity of cranial ornaments among late-branching ornithopods, with important implications regarding the specific function and convergent evolution of the hollow supracranial crest within hadrosauroids.

## 2. Material and Methods

### 2.1. Individual Assignment of Material

The specific individual assignment of the new material was confirmed on the basis of the taphonomic condition and osteological association of the retrieved bone elements. The taphonomic condition of the material in situ gradually emerged during the preparation of the plaster jacket, and finally revealed an incomplete, semi-articulated skull belonging to a single individual. The right half of the partial facial skeleton is essentially articulated, whereas the left half is largely disarticulated and moderately damaged, along with the shedding of teeth from the left maxilla and dentary. The reason for this taphonomic condition is predicted to be attributed to the sufficient air exposure of the left half of the skull after soft tissue decay, with the influences of weathering and biological mechanisms during the hydraulic transportation and sorting [27] (see Supplementary Materials). Although the braincase is missing from the skull in situ, a potential substitute was found ~80 m to the southwest of the partial skull. We regard the isolated braincase as belonging to CIP V0002 because of the perfect correspondence between osseous contacts, such as the deeply excavated anterior platform of the frontal fitting well with the strongly convex nasal posteroventral facet.

### 2.2. Field and Laboratory Preparation of Material

**Figure 1:**
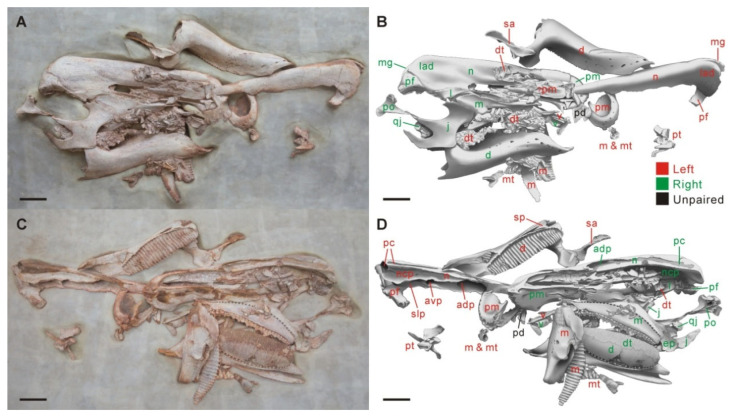
Photograph (**A**) and drawing (**B**) of the two-dimensionally displayed partial skull of *Qianjiangsaurus changshengi* except for the braincase (CIP V0002) from one side of the plaster casing. Photograph (**C**) and drawing (**D**) of the same skull from the other side of the plaster casing. Scale bar equals 10 cm.

The partial skull excluding the isolated braincase (field number: 24QZ-DDLZ2) was collected from a small quarry that is approximately 1.8 m long and 1.2 m wide, and was encased with the matrix in a plaster jacket (~1.32 m × 0.82 m). Preparation at the lab was conducted by technicians under the microscope using pneumatic engraving pens, chip paint brushes and Eastman Butvar B-72 polyvinyl butyral resin. The asymmetric preservation of the facial skeleton (i.e., articulated right half versus damaged, disarticulated left half) and the presence of numerous scattered teeth alongside the skull made full preparation difficult. Therefore, the partial skull within the jacket was finally prepared for the largely two-dimensional display on an openable, two-sided plaster casing (Figure 1).

### 2.3. Three-Dimensional (3D) Reconstruction of the Endonasal Morphology

To visualize its internal structure, the main body of the left nasal was removed from the plaster casing and scanned using the industrial computed tomography (CT) system (mi-CT 450ICT) at the laboratory of the Institute of Vertebrate Paleontology and Paleoanthropology, Chinese Academy of Sciences (CAS), Beijing, China. We specified the following CT parameter settings: slice thickness = 160 μm, source voltage = 430 kV, and source current = 1.5 mA. Raw projection data were produced by CT scans, and were later reconstructed using the digitized software developed by the Institute of High Energy Physics, CAS, Beijing, China. The resulting image stacks were exported as TIFF files and subsequently imported into Dragonfly 2024.01 [28] for image processing, where the nasal bone and endonasal cavity were digitally extracted using segmentation tools. The STL files were exported from Dragonfly and then imported to Blender 4.2 [29] for 3D reconstruction and visualization, where the digitized nasal and endonasal morphology was refined using modeling and sculpting tools. Finally, the digitized left nasal and endonasal cavity were mirrored to produce paired elements, providing an overall morphology for study.

### 2.4. Phylogenetic Analysis

To re-evaluate the systematic position of *Qianjiangsaurus changshengi* within Hadrosauroidea after a supplement to the previously unknown cranial information, a cladistic analysis using maximum parsimony was conducted based on an updated data matrix, the character and taxon lists of which entirely follow Dai et al. [26] (see Supplementary Materials). The data matrix was modified in Mesquite 3.10 [30], and was later analyzed in TNT 1.1 [31], with a maximum of 10,000 trees held in memory. A traditional search specifying a random seed of 1 and 5000 replicates for Wagner trees was performed using the swapping algorithm of tree bisection reconnection that supplied 100 trees saved per replicate. Here we use “early-branching hadrosauroids” to denote non-hadrosaurid hadrosauroids.

### 2.5. Calculations of Resonant Frequencies on the Nasal Cavity

Hypertrophied nasal passages in lambeosaurines are thought to have functioned as acoustic resonators in life [17]. Weishampel [19] first calculated resonant frequencies for the tube-like nasal cavity of *Parasaurolophus*, but argued that the more complex nasal cavities of variable shape in other lambeosaurines may confine the application of this arithmetic approach. Despite this, the calculations suggested by Weishampel [19] can effectively construct a comparative framework to evaluate the relative acoustic potential of the nasal cavity among non-*Parasaurolophus* hadrosauroids (see Supplementary Materials for further details). Accordingly, our work does not attempt to reconstruct exact vocal frequencies, but instead explores how differences in nasal cavity morphology theoretically influence the resonant regimes and functions of airways among hadrosauroids.

Fourteen hadrosauroid species represented by 17 individuals were included in the comparative acoustic analysis on resonant frequencies of nasal cavities, and two of the lambeosaurine species involve multiple ontogenetic stages. Airway lengths of nasal cavities were measured using ImageJ 1.54g from photographs and CT reconstructions that include scale bars. During the measurement in lateral view, the posteroventral extent of the nasal cavity was presumably terminated near the paired contacts between the jugal and maxilla, where the internal nares or choanae occur along the palatal region [32]. We suppose that sufficient resonance of the nasal cavity was achieved as air was passed up from the vocal organ (e.g., the throat) during exhalation, rather than during inhalation. Therefore, the starting point of the nasal cavity airway for length measurements is limited to the region of the internal naris. For lambeosaurines, nasal cavity measurements were taken from the literature on 3D skull reconstruction, such as Evans et al. [9]. For the skull of *Qianjiangsaurus changshengi*, in addition to the primary airway between the external nares and nasal cavity proper, the lengths of the other airway system throughout the anterodorsal and anteroventral passages, respectively, were measured separately. The airways within the nasal cavity of *Q. changshengi* were modeled as open-ended tubes, with the lengths measured from the internal naris to either the anterior openings of the endonasal passages or the posterior extremity of the external naris. The airways of lambeosaurines via the lateral diverticula were modeled as closed-ended tubes, measured from the internal naris to the closed extremity of each diverticulum. When multiple airway systems of the nasal cavity acted together as an integrated acoustic resonator, we considered a range of resonant frequencies in between (see Supplementary Materials).

### 2.6. Anatomical Abbreviations

adp, anterodorsal passage; alp, alar process; ap, anterior platform; avp, anteroventral passage; bo, basioccipital; bpp, basipterygoid process; bsp, basisphenoid; cmc, common median chamber; csn, contact surface for the nasal; d, dentary; dt, dentary teeth; ep, ectopterygoid; ex, exoccipital; f, frontal; hnc, hollow nasal crest; j, jugal; l, lacrimal; lad, lateral dome; ld, lateral diverticulum; lsp, laterosphenoid; m, maxilla; mg, medial groove; mt, maxillary teeth; n, nasal; ncp, nasal cavity proper; om, orbital margin; osp, orbitosphenoid; p, parietal; pc, posterior chamber; pd, predentary; pf, prefrontal; pm, premaxilla; po, postorbital; pro, prootic; pt, pterygoid; qj, quadratojugal; sa, surangular; slp, special lateral passage; so, supraoccipital; sp, splenial; v, vomer.

### 2.7. Institutional Abbreviations

CIP, Chongqing Institute of Paleontology, Chongqing, China; CLGRP, Chongqing Laboratory of Geoheritage Research and Protection, Chongqing Bureau of Geological and Mineral Resource Exploration and Development, Chongqing, China.

## 3. Systematic Paleontology

Dinosauria Owen [33]

Ornithischia Seeley [34]

Ornithopoda Marsh [35]

Hadrosauriformes Sereno [36] *sensu* Sereno [37]

Hadrosauroidea Cope [38] *sensu* Sereno [37]

*Qianjiangsaurus changshengi* Dai et al. [26]

### 3.1. Holotype

CLGRP V00016, an incomplete, partially articulated skeleton, including only two skull elements (i.e., the predentary and left dentary), four dorsal vertebrae, the sacrum, anterior and middle caudal series with chevrons, the right ilium, the left pubis, the paired ischia, the left femur, the paired tibiae, the left fibula, the left astragalus, left metatarsals II and III, and right metatarsal IV.

### 3.2. Referred Material

CIP V0002, a partial, semi-articulated skull missing part of the facial skeleton and lower jaw.

### 3.3. Locality and Horizon

Zhengyang area, Qianjiang District, Chongqing Municipality, southwest China; thick layer of reddish brown and purplish gray siltstone to sandstone (lithofacies change) with calcareous nodules and small pebbles, from the uppermost part of the Zhengyang Formation (see Supplementary Materials); late stage of the Late Cretaceous (~84 Ma: unpublished data by J. Wang) [26].

### 3.4. Revised Diagnosis

Medium-sized non-hadrosaurid hadrosauroid (~8 m long in presumable adults) with the following unique combination of features (probable autopomorphies*): strongly posterodorsally reflected, lip-shaped premaxillary oral margin; absence of an elevated accessory fossa in the premaxillary prenarial region; greatly anteroposteriorly elongate but extremely dorsoventrally constricted external naris enclosed by the premaxilla and nasal; anterior half of the nasal ventrally excavated by a deeply arcuate fossa near midlength; dorsoventral thickening of the nasal along its anterior process and contact surface for the counterpart, with a nearly straight dorsomedial margin of the bone above the external naris*; incipiently developed, helmet-shaped hollow supracranial crest exclusively formed by the posterior quarter of the paired nasals, showing a narrow medial groove and a wide lateral dome along each half of the dorsal crest*; presence of paired accessory endonasal cavities directly dorsal to the nasal cavity proper, each consisting of an elongate anterodorsal passage, a short, slightly curved anteroventral passage, their common posterior chamber, and a special subtrapezoidal lateral passage connecting the posterior chamber with the nasal cavity proper, together along the posterior half of the nasal*; truncated but elevated anterodorsal process of the maxilla relative to the anteroventral process; markedly anteroposteriorly expanded, roughly parallelogram-shaped ectocranial surface of the maxillary dorsal ramus anterior to the dorsoventrally deep, regularly diamond-shaped contact surface for the jugal*; dorsoventrally expanded, bluntly rounded anterior process of the jugal with moderately recurved dorsal and ventral convexities; slender, hook-like posterodorsal process of the jugal with a nearly straight posterior edge; orbital region of the jugal wider than the infratemporal one; dorsoventrally high, laterodorsally convex anteroposteriorly ectocranial region of the prefrontal laterally overlapping the posterior portion of the hollow nasal crest, with a large, dorsally positioned, oval foramen and a widely arcuate orbital margin marked by a shallow, anteriorly inclined, slightly sinuous groove*; lateral contact between the prefrontal and lacrimal largely concealed by the overgrowth of the nasal*; frontal contributing to the orbital margin; relatively short, deeply excavated, steeply ventrally sloping frontal anterior platform for the base of the nasal crest that is anteromedially–posterolaterally oriented, with an elevated, slightly convex posteromedial margin; elongate, nearly straight anterolateral processes of the parietal; small, ventrally tapering process of each half of the basisphenoid occurring between the bases of the basipterygoid and alar processes*; transversely broad, U-shaped predentary bearing smoothly arcuate anterolateral borders; subtrapezoidal denticles along the oral margin of the predentary; maximum of five teeth per alveolus (including three functional ones) in the middle of the dentary tooth battery; nearly vertical coronoid process of the dentary, with a subcircular, strongly anteroposteriorly expanded apex exhibiting a more prominent anterior edge; dentary tooth crowns lingually ornamented with a slightly distally offset primary ridge, a short secondary ridge mesially and a slender faint ridge distally; greatly transversely expanded neural spine of the dorsal vertebra bearing slightly dorsally diverging lateral sides; deeply concave posterodorsal margin of the central plate of the ilium above the supraacetabular process; fan-shaped prepubic process of the pubis remarkably anteroposteriorly shortened and dorsoventrally expanded, with a length/height ratio of ~0.79*; and ischial shaft nearly parallel with the long axis of the pubic peduncle of the ischium.

## 4. Supplementary Skull Description and Comparisons

**Figure 2:**
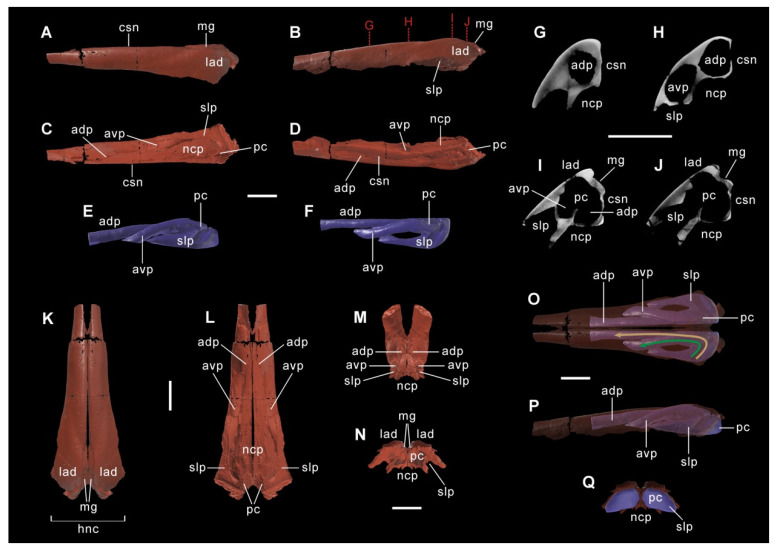
Images on 3D reconstruction of the main body of the left nasal and its accessory internal cavity by CT scans. 3D model of the main body of the left nasal in dorsal (**A**), lateral (**B**), ventral (**C**) and medial (**D**) views. 3D model of the left accessory endonasal cavity in lateral (**E**) and dorsal (**F**) views. CT cross sections along the posterior half of the left nasal in anteroposterior sequence (**G**–**J**). 3D model of the combined main body of the left nasal and its mirror copy in dorsal (**K**), ventral (**L**), anteroventral (**M**) and posterior (**N**) views. Translucent 3D model of the combined main body of the left nasal and its mirror copy showing the paired accessory endonasal cavities in dorsal (**O**), left lateral (**P**) and posterior (**Q**) views. The airways via the anterodorsal and anteroventral passages are indicated by the golden and green arrows, respectively. Scale bar equals 10 cm.

**Figure 3:**
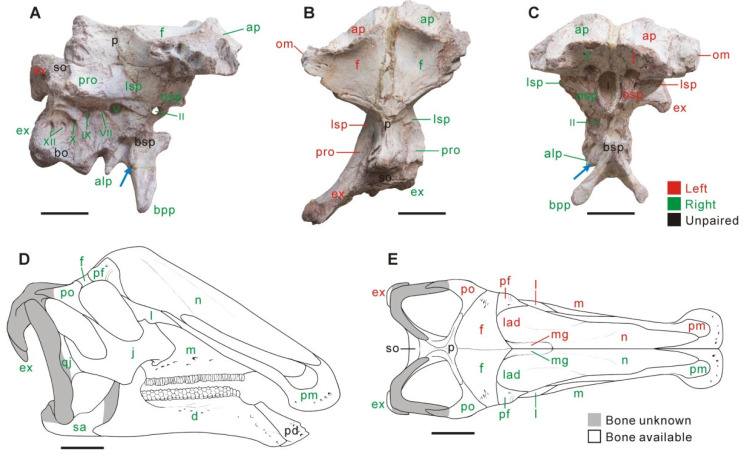
Partial braincase of *Qianjiangsaurus changshengi* (CIP V0002) in right lateral (**A**), dorsal (**B**) and anterior (**C**) views, along with the skull reconstruction of line drawing for the taxon in right lateral (**D**) and dorsal (**E**) views. The blue arrow indicates the ventrally tapering process of the basisphenoid. Scale bar equals 5 cm for (**A**–**C**) and 10 cm for (**D**,**E**).

The skull CIP V0002 probably pertains to a subadult individual, based on its relatively large size and obscure calvarial sutures that remain slightly open. Linear measurement comparisons of the overlapping skull elements between CIP V0002 and the holotype CLGRP V00016, which show slightly small size relative to the holotype at the presumably adult stage, are listed in Supplementary Materials. Here we provide a concise comparative description of this specimen to supplement important skull characters of *Qianjiangsaurus changshengi* not stated by Dai et al. [26], especially those of great diagnostic and phylogenetic significance, and those that probably reveal phenotypic and functional convergence with late-branching lambeosaurines (Figure 1, Figure 2 and Figure 3; Supplementary Materials, Figures S4–S13). Terminology for skull and nasal cavity anatomy follows Weishampel [8], Evans [12,16] and Xing et al. [39].

### 4.1. Facial Skeleton

The paired premaxillae feature a transversely widened, duck-billed oral region that gradually constricts posteriorly in advance of the external nares. The premaxillary oral margin strongly reflects posterodorsally to form a thick, lip-shaped structure, very similar to the condition in *Edmontosaurus* and *Shantungosaurus* [39] but strikingly contrasts with the gently swollen or upturned equivalent in other hadrosauroids [40]. Posterior to the oral margin, the prenarial part of the premaxilla is relatively smooth, and lacks the slightly elevated accessory fossa and foramen common among saurolophines [41,42]. A greatly anteroposteriorly elongate but particularly dorsoventrally narrow, spindle-shaped external naris, entirely enclosed by the premaxilla and nasal, is preserved on the right side of the skull. This is very similar to the narial shape in *Prosaurolophus* and *Saurolophus* [40], but is distinct from the relatively dorsoventrally deep, suboval or teardrop-like external naris seen in many other ornithopods [22,43,44], and the simplified external naris exclusively encircled by the premaxilla in most lambeosaurines [6,7,12,15]. In CIP V0002, the nasal forms the posterior extremity of the external naris, which is located directly dorsal to the anterolateral contact of the lacrimal and maxilla. The nasal is dorsoventrally thickened along its elongate anterior process and contact surface for its counterpart, and has a nearly straight dorsomedial edge above the external naris. The robust, finger-shaped anterior process of the nasal extends to the prenarial region of the snout. Following the dorsoventral thickening of the anterior process, the posterior quarter of the nasal is slightly overgrown to exclusively form half of the relatively low, helmet-shaped supracranial crest, which displays a narrow medial groove and a wide lateral dome in dorsal view (Figure 1 and Figure 2). In ventral view, the anterior half of the nasal is excavated by a prominent, deeply arcuate fossa near midlength. Posterior to this fossa, two large, ovate foramina along the ventromedial side of the posterior half of the nasal anterior process serve as the anterior openings of a long, tube-like endonasal passage and another slightly short, more posterolaterally and ventrally positioned endonasal passage of similar shape. Owing to the slight breakage of the enclosed posterior end of the left nasal, a common posterior chamber at the convergence of the two passages, as well as a special lateral passage connecting the chamber with the underlying nasal cavity proper, can be identified by direct observation (Figure 1; Supplementary Materials, Figure S7). This condition clearly validates the presence of paired additional internal cavities within the nasal crest (i.e., the hollow nasal crest). The special lateral passage is incompletely separated from the nasal cavity proper by a lateroventrally directed lamina.

The maxilla has an elevated anterodorsal process, as in *Eotrachodon* and saurolophines; however, the process is proportionally shorter than that in the latter taxa [40,45]. A dorsoventrally deep, regularly diamond-shaped sutural surface for the jugal occurs along the middle portion of the maxillary lateral surface, which closely resembles the condition in corythosaurian lambeosaurines (consisting of the tribes Parasaurolophini and Lambeosaurini) [12,40,46]. Interestingly, the dorsal ramus of the maxilla is laterally occupied by an anteroposteriorly expanded, roughly parallelogram-shaped ectocranial surface immediately anterior to the dorsal half of the sutural facet for the jugal, and the ventral extremity of the sutural facet lies a short distance from the more posteriorly positioned ectopterygoid ridge. By contrast, in corythosaurian lambeosaurines, the lateral surface of the dorsal ramus is largely formed by the dorsal half of the sutural facet for the jugal, and the ventral extremity of the sutural facet abuts the anterior end of the ectopterygoid ridge [12,15,40]. As in most early-branching hadrosauroids, the skull CIP V0002 has a relatively thin anterior part of the ectopterygoid ridge, a series of anteroposteriorly scattered maxillary lateral foramina of variable size below the sutural facet for the jugal, and a slender, hook-like posterodorsal process of the jugal with a nearly straight posterior edge [47,48,49]. Quite similar to the condition in lambeosaurines [40], the anterior process of the jugal is dorsoventrally expanded and bluntly rounded, with moderately recurved dorsal and ventral convexities. However, the anteroventral expansion of the process is more exaggerated than that in lambeosaurines, forming a nearly right-angled protrusion. The dorsoventrally high, laterodorsally convex anteroposteriorly ectocranial part of the prefrontal laterally overlaps the posteroventral region of the hollow nasal crest, which is generally comparable to but essentially different from the sharp, strongly elevated medial ridge of the prefrontal to laterally support the base of the hollow crest (consisting of the nasals and premaxillae) in most lambeosaurines, such as *Jaxartosaurus* and *Hypacrosaurus* [12,46]. The thick, widely arcuate orbital margin of the prefrontal is laterally ornamented by a shallow, slightly sinuous groove that is anteriorly inclined. In lateral view, the prefrontal contact with the lacrimal is nearly completely hidden by the ventral overgrowth of the posterior plate of the nasal. It is unlikely that the prefrontal meets the premaxillary posteroventral process, due to the intrusion of the enlarged nasal. The anteromedial process of the postorbital does not strongly protrude dorsally, as in non-hadrosaurid hadrosauroids and most saurolophines [40].

### 4.2. Braincase

The frontal greatly contributes to the orbital margin, similar to the condition in early-branching hadrosauroids, including *Probactrosaurus* and *Tethyshadros* [50,51]. In dorsal view, the paired frontals comprise the anterior platform for the hollow nasal crest. The anterior platform is deeply excavated and steeply inclined ventrally. It is comparable to those of many adult late-branching lambeosaurines [7,12,46]. In CIP V0002, the platform is ~35% as long as the ectocranial surface of the frontals, and is proportionally much shorter than that in most presumably adult lambeosaurines, where this structure consists of the paired prefrontal and frontals [7,12,52]. Each half of the anterior platform in CIP V0002 is anteromedially–posterolaterally oriented and defined by an elevated, slightly convex posteromedial margin. The gently concave ectocranial surface of each frontal is anteroposteriorly longer than mediolaterally wide. The parietal has a pair of elongate, nearly straight anterolateral processes, as in lambeosaurines. This contrasts with the relatively short, laterally curved equivalent processes seen in many non-lambeosaurine hadrosauroids, such as *Levnesovia* and *Acristavus* [23,39,48,50]. The sagittal crest of the parietal splits into two posterolateral ridges along its posterior half, and the ridges form an angle of ~38° with each other. As in *Eotrachodon* and hadrosaurids [45,53], the basisphenoid does not contribute to the foramen for the trigeminal nerve (CN V). A pair of relatively small, ventrally tapering processes occurs between the paired alar and basipterygoid processes of the basisphenoid, previously unreported in other ornithopods (Figure 3A,C).

### 4.3. Accessory Endonasal Cavities

3D reconstruction using CT scans and digitized image processing further corroborates the presence of additional internal cavities within the paired nasals. There is a pair of accessory endonasal cavities separated from each other by the medial mutual contact of the left and right nasals, directly dorsal to the nasal cavity proper that follows behind the paired external nares, each of which is encircled by the premaxilla and nasal. Each accessory endonasal cavity occurs along the posterior half of the nasal, and consists of an elongate anterodorsal passage, a short, slightly curved anteroventral passage, their common posterior chamber and a special lateral passage (subtrapezoidal in lateral outline) that connects the posterior chamber with the nasal cavity proper (Figure 2E,F). The outlines of the two internal passages are vaguely visible along the laterodorsal surface of the right nasal, as indicated by two shallow grooves posterodorsal to the external naris, which gradually converge towards the helmet-shaped nasal crest. Thus, two sets of main airways for breathing are independently present but closely associated around the paired nasals and neighboring bones: one is the traditional form that extends directly from the elongate paired external nares to the nasal cavity proper, similar to the condition in other non-lambeosaurine ornithopods; the other is a symmetrical complex positioned dorsal to the traditional airway, running from two paired anterior openings medial to the posterodorsal margin of the external nares to the posterior region of the nasal cavity proper throughout the accessory endonasal cavities. The accessory endonasal cavity is a novel structure never reported previously, and is probably homologous with the solid internal region of the posterior half of the nasal in early-branching hadrosauroids and saurolophines. By contrast, the nasal cavity and airway in many late-branching lambeosaurines (e.g., corythosaurians) are extremely hypertrophied, housed by the enlarged premaxillae and nasals, and consist of a pair of sigmoid or intertwined loops, a supraorbital common median chamber at the posterior convergence of the loops, and two closed-ended lateral diverticula branching off from the chamber [8,9,16,54]; the s-loop and lateral diverticulum are generally equivalent to the nasal vestibule, and the common median chamber is largely homologous with the nasal cavity proper [16,17]. Thus, the hollow supracranial crests with endocranial airways exhibited by both *Qianjiangsaurus* and corythosaurian lambeosaurines essentially differ in osteological configurations and homology.

### 4.4. Lower Jaw

The coronoid process of the dentary bears a subcircular, strongly anteroposteriorly expanded apex, the anterior edge of which is more prominent than the posterior edge. This condition is comparable to that seen in several non-hadrosaurid hadrosauroids and many lambeosaurines, such as *Nanningosaurus* and *Amurosaurus* [11,40,46,55]. The symphysial process of the dentary is laterally perforated by a series of small, subovate foramina. There are no foramina along the lateral sides of the anterodorsal process and laterodorsal flange of the surangular, in contrast to the condition in some early-branching hadrosauriforms such as *Jinzhousaurus* and *Eolambia*, where the accessory foramina of the lateral surangular are visible [56,57].

### 4.5. Dentition

Each maxillary tooth crown is evenly separated by a straight primary ridge along its labial surface, with no evidence of any faint ridge. There are one or two functional teeth per alveolus along the entire occlusal surface of the maxillary dental battery, as in many early-branching hadrosauroids, including *Gobihadros* and *Gilmoreosaurus* [49,58]. In CIP V0002, the upper and lower dental batteries contain 30 and 28 tooth positions, respectively.

## 5. Phylogenetic Reassessment

**Figure 4:**
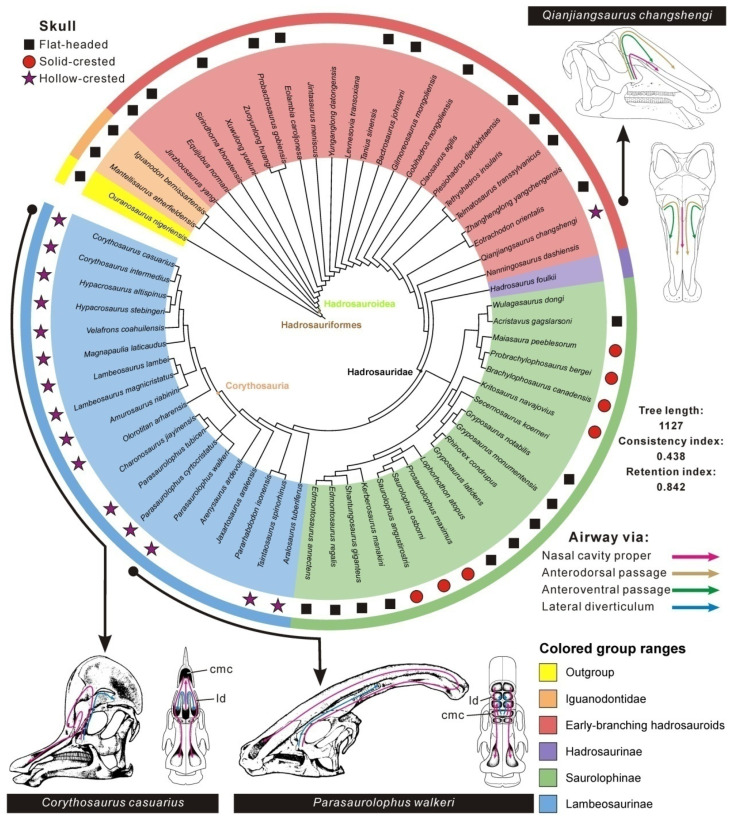
Circular cladogram of Hadrosauroidea derived from the strict consensus of two MPTs using maximum parsimony, displaying a particularly highly nested position of *Qianjiangsaurus changshengi* within the phylogenetic topology of non-hadrosaurid hadrosauroids, as well as revealing a structural diversity of hollow supracranial crests among non-saurolophine hadrosauroids.

After the incorporation of cranial data for *Qianjiangsaurus changshengi*, the updated phylogenetic analysis of Hadrosauroidea resulted in two most parsimonious trees (MPTs) of 1126 steps each (consistency index = 0.439, retention index = 0.842). Compared with the phylogenetic topology shown in Dai et al. [26], *Q. changshengi* is no longer recovered as the sister taxon to *Plesiohadros djadokhtaensis* in the strict consensus, and is instead positioned higher on the tree than the clade of *Zhanghenglong yangchengensis* + *Eotrachodon orientalis* but just below *Nanningosaurus dashiensis* directly outside of Hadrosauridae (Figure 4). Therefore, the current analysis still places *Q. changshengi* in a highly nested position of the tree topology regarding non-hadrosaurid hadrosauroids, which is very similar to the result of Dai et al. [26]. Our cladistic analysis recovers a sister-taxon relationship between *N. dashiensis* and Hadrosauridae. The phylogenetic framework of Hadrosauridae does not differ significantly from that in Prieto-Márquez [40] and Dai et al. [26], where this taxon is divided into the earliest-branching *Hadrosaurus foulkii* (i.e., Hadrosaurinae), Saurolophinae and Lambeosaurinae. Furthermore, the clade of *Telmatosaurus transsylvanicus* + *Tethyshadros insularius* is postulated to be later-diverging than the lineage of *P. djadokhtaensis* but earlier-diverging than the clade consisting of *Z. yangchengensis* and *E. orientalis*; *Claosaurus agilis* is depicted as more closely related to *P. djadokhtaensis* than to *Gobihadros mongoliensis*, *Gilmoreosaurus mongoliensis* and *Bactrosaurus johnsoni*. As shown in Figure 4, the phylogenetic framework reveals a limited distribution of sold crests within Saurolophinae, as well as an expansion of the distribution range of hollow crests from Lambeosaurinae to early-branching hadrosauroids.

## 6. Discussion

### 6.1. Taxonomic Status

The holotype and newly collected skull of *Qianjiangsaurus changshengi* together display numerous plesiomorphic features that are typical of non-hadrosaurid hadrosauroids but rarely reported in hadrosaurids. These features include: a smooth anterior portion of the premaxilla between the open external naris and thickened oral margin; anteroposteriorly aligned, scattered lateral foramina of the maxilla numbering at least seven; the markedly posteroventrally inclined ectopterygoid ridge and shelf of the maxilla; a maxilla having no more than 32 alveoli and one or two occluding teeth per alveolus; a slender, hook-like posterodorsal process of the jugal with a nearly straight posterior margin; a sagittal crest that splits into two posterolateral ridges along the posterior half of the parietal; an extensive contribution of the frontal to the orbital margin; a transversely broad predentary with a U-shaped dorsal outline and widely arched anterolateral margins; a dentary having no more than 30 alveoli; a coronoid process forming a right angle with the dorsal edge of the dentary ramus; the dentary tooth crowns lingually ornamented with a slightly distally displaced primary ridge from the midline, a short secondary ridge mesially and a slender faint ridge distally; a sacrum composed of no more than seven fused vertebrae; an anterior blade of the prepubic process of the pubis expanded more dorsally than ventrally; and a nearly right-angled, foot-like protuberance of the distal ischium that lacks anterior curvature [22,40,50,57,58,59]. The presence of these features is inclined to indicate that *Q. changshengi* is affiliated with early-branching hadrosauroids outside of Hadrosauridae. This comparative osteology, along with our updated phylogenetic framework (see above), further demonstrates that *Q. changshengi* is a late-branching non-hadrosaurid hadrosauroid, although this taxon shares a series of convergent craniofacial analogs with corythosaurian lambeosaurines.

### 6.2. Comparative Resonant Frequencies of Nasal Cavities Among Hadrosauroids

**Figure 5:**
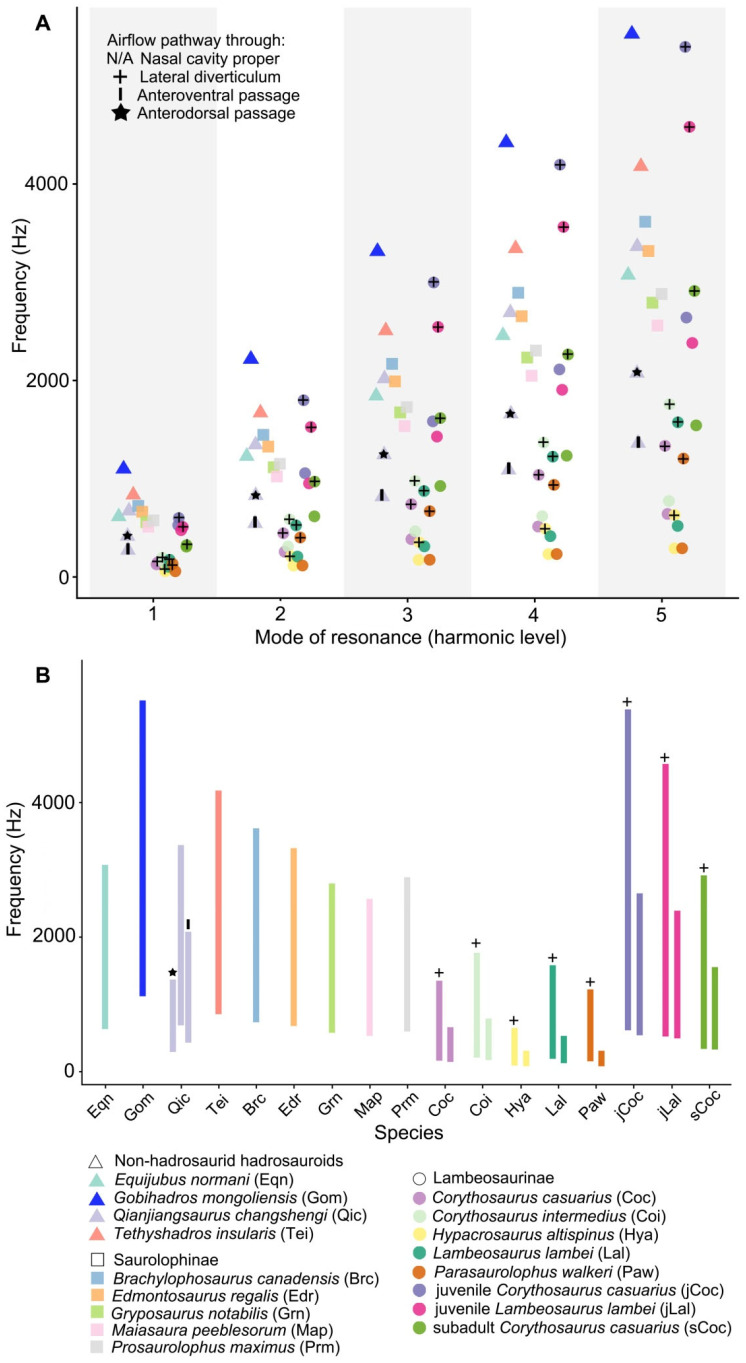
Scatter plot (**A**) and bar chart (**B**) showing differentiations of resonant frequencies of nasal cavities among hadrosauroids under various harmonic levels and airflow pathways, respectively.

Comparisons of estimated resonant frequencies of nasal cavities among hadrosauroids reveal differences in tone ranges of vocal outputs, particularly at the lowest vocal frequencies, assuming that the vocal organ produced standing waves of sound from the fundamental tone (i.e., the 1st harmonic) to the 5th harmonic (Figure 5). Five adult lambeosaurines exhibit the lowest range of predicted resonant frequencies, irrespective of the chosen harmonic (e.g., approximately 58–154 Hz for the 1st harmonic); for each lambeosaurine, the resonant frequency of the airway via the lateral diverticulum is slightly higher than that through the common median chamber (i.e., the nasal cavity proper) (Figure 5A; Supplementary Materials, Tables S2 and S3). By contrast, non-hadrosaurid hadrosauroids and saurolophines show broadly overlapping ranges of resonant frequencies, regarding the primary airway via the nasal cavity proper; a comparison of estimated frequency ranges, along with the change of the harmonic level, indicates no statistically significant difference between the two groups (*t*-test, *p* = 0.7476). Conversely, the frequency range difference between adult lambeosaurines and the other two groups is statistically significant (*p* = 0.0001–0.0004 for non-hadrosaurid hadrosauroids; *p* = 0.0001 for saurolophines), following the increase of the harmonic level for resonance (Supplementary Materials, Table S5). These statistical comparisons of frequency ranges suggest that adult lambeosaurines were capable of vocalizations with extremely low resonant frequencies relative to the other two groups (even though the airway via the lateral diverticulum would have played a vital role). However, non-lambeosaurine hadrosauroids except *Qianjiangsaurus changshengi* reveal the higher ranges of vocal frequencies, which become more distinct with increasing harmonic level for nasal cavity resonance.

The nasal cavity of *Qianjiangsaurus changshengi* probably contains three distinct airflow pathways during exhalation: the primary pathway directly via the nasal cavity proper, and the additional pathways via the anterodorsal and anteroventral passages. The primary airflow pathway would produce relatively higher resonant frequencies (~672–3364 Hz for the 1st to 5th harmonics), largely overlapping with the estimated frequency ranges of other non-hadrosaurid hadrosauroids and saurolophines (Figure 5B). By contrast, the additional airflow pathways via the two passages would generate lower ranges of resonant frequencies (~272–2761 Hz and ~415–2074 Hz for the 1st to 5th harmonics, respectively), which partially overlap with the frequency domain of adult lambeosaurines. This frequency overlap indicates that *Q. changshengi* may have been capable of producing low-frequency sounds comparable to the frequency ranges inferred for adult lambeosaurines throughout selective airflow pathways. Such flexibility may have been controlled by soft-tissue structures analogous to muscular or valvular systems in extant vertebrates, such as nasal valves of mammals that could adjust the major direction of the airflow toward either the nasal vestibule or the dorsal region of paranasal sinuses [60]. The structures possibly in *Q. changshengi* would have allowed a selective major airflow route or simultaneous airflow routes of equal importance that enabled the change of acoustic frequencies and modulation of vocal pitches by switching to the effective resonant airway or airway combination. The two airflow pathways via the anterodorsal and anteroventral passages within the accessory endonasal cavity of *Q. changshengi* exhibit the approaching values regarding the resonant frequency (Supplementary Materials, Table S2), and the interference of sound waves within this cavity would occur to produce acoustic beats (otherwise known as beat frequencies), which could be expressed as variable amplitude undulations and increasing spectral complexity [61].

### 6.3. Functional Implications of Accessory Endonasal Cavities

The ability to make low-frequency vocalizations is long regarded to have been evolutionary advantageous for lambeosaurine dinosaurs [9,19,20]. This is largely because sound waves with frequencies below 400 Hz can efficiently propagate over long distances and are less affected by habitat structure [62,63]. Low-frequency vocalizations can also play an important anti-predator role in some extant gregarious herbivores. For example, in alcelaphine bovids, such as wildebeest (*Connochaetes*) and hartebeest (*Alcelaphus*), fairly low-frequency sounds may be outside the hearing range of many carnivores, reducing survival risk to be detected by predators [25]. Taken together, vocalization with relatively low frequencies is a significant survival strategy for some extinct and extant herbivores, which may aid in the long-distance signaling and avoidance of predators.

Comparisons of endocranial airway morphology and resulting resonant frequencies of nasal cavities imply that *Qianjiangsaurus changshengi* and corythosaurian lambeosaurines may have used their independently derived hollow supracranial crests with different endocranial configurations to perform similar vocalization functions, within the scope of Hadrosauroidea. In adult lambeosaurines, the elongation and hypertrophy of the nasal cavity (essentially homologous with the nasal vestibule and nasal cavity proper) provided a structural basis for the production of low-frequency sounds, with a total resonant frequency range of ~58–1758 Hz spanning the 1st to 5th harmonics (Figure 5). By contrast, *Q. changshengi* would have achieved low-frequency vocalizations based on a complex additional airway system within the paired nasals, located just above the traditional endocranial airway directly via the nasal cavity proper (Figure 2). This novel airway system consists of paired accessory endonasal cavities, each of which includes two partly overlapping airflow pathways facilitating similar low-frequency vocalizations (e.g., ~272 Hz and ~415 Hz at the level of the 1st harmonic for the two airflow pathways via the anterodorsal and anteroventral passages, respectively). These inferences reveal that the production of low-frequency sounds within Hadrosauroidea is not tied to a single cranial configuration with the constant endocranial morphology, but could have been realized throughout alternative internal structures regarding the nasal cavity under comparable functional constraints. The hollow supracranial crest in the presumably subadult individual of *Q. changshengi* is relatively low, quite comparable to the incipiently developed analog in juvenile forms of lambeosaurin lambeosaurines [9,12,54]. Therefore, the hollow nasal crest with the novel accessory endonasal cavities in *Q. changshengi* appears to behaviorally function as a secondary acoustic resonator for the low-frequency switch of vocalizations, which would be very helpful in safer, more efficient social communication with conspecific fellows and coeval late-branching lambeosaurines. The beat frequencies produced by the two airflow pathways within the accessory endonasal cavity of *Q. changshengi* may have led to a diversity of low-frequency sounds in pitch and volume.

### 6.4. Evolutionary Mechanisms of Cranial Convergence

Our study presents a series of salient characters of the non-hadrosaurid hadrosauroid *Qianjiangsaurus changshengi* that distinctly resemble some apomorphic features common among corythosaurian lambeosaurines. These analogous characters in *Q. changshengi* are: (1) the overgrowth of nasals serving as a hollow supracranial crest with a complex internal cavity; (2) a deeply excavated, steeply sloping anterior platform of the frontal for supporting the hollow crest; (3) a strongly elevated ectocranial region of the prefrontal that laterally limits the hollow crest; (4) a dorsoventrally deep, regularly diamond-shaped maxillary contact surface for the jugal; (5) a dorsoventrally expanded, bluntly rounded anterior process of the jugal with moderately recurved dorsal and ventral convexities; (6) a subcircular, strongly anteroposteriorly expanded apex of the dentary coronoid process exhibiting a more prominent anterior edge; and (7) elongate, nearly straight paired anterolateral processes of the parietal. This is the first report of multiple typical skull characters of late-branching lambeosaurines independently and intensively emerging in another ornithopod taxon outside Lambeosaurinae. Amongst these listed characters, the first three are long regarded as associated with the development of the tube-like or helmet-shaped hollow supracranial crest possibly leading to vocal communication via acoustic resonance [9,12,16].

In evolutionary biology, convergent evolution is defined as the process whereby distinct taxa or lineages independently evolve analogous features, structures and functions, likely to adapt to similar necessities (e.g., environments). The subject has fascinated evolutionary biologists and paleontologists for centuries. The shared patterns of comparable skull characters, together with comparisons of resonant frequencies on the nasal cavity among hadrosauroids, reveal a striking morphological and functional convergence between *Qianjiangsaurus changshengi* and late-branching lambeosaurines.

Regarding the evolutionary mechanisms, skull characters 1–3 above may have undergone convergent adaptive evolution, selected for abilities of low-frequency vocal communication. For example, the nasal overgrowth may have been favored under natural selection for an expanded acoustic resonating cavity that would have resulted in efficient sound propagation with low frequencies. While having no direct contribution to such adaptive functions, skull characters 4–7 likely experienced convergent changes due to their shared developmental constraints with the traits under natural selection. Such coevolution of related features is often attributed to the shared developmental regulation of pleiotropic genes. Like other complex morphological characters, the processes of craniofacial morphogenesis and osteogenesis are realized by a multilayered regulatory network involving upstream regulator genes [64,65]. These regulatory genes are developmentally pleiotropic, as their changes may influence multiple craniofacial features, including our focal morphological characters. Hence, the adaptive change of these genes for structural support of low-frequency vocal communication may also lead to the concerted evolutionary changes of seemingly functionally unrelated but morphologically related characters within the same development module. Previous studies on primate limb morphology and other traits have also supported the prevalence of pleiotropy and its phenotypic effects in development [66,67]. In conclusion, the series of trait convergence observed in skulls of *Qianjiangsaurus changshengi* and late-branching lambeosaurines can be effectively explained by adaptive evolution under similar pressures of natural selection, combined with developmental constraints due to gene pleiotropy.

## 7. Conclusions

*Qianjiangsaurus changshengi* is previously known only from a partial skeleton mostly constituted by the postcranium, leading to unresolved issues concerning its cranial morphology and function. The significant discovery of a well-preserved skull reveals that this early-diverging hadrosauroid exhibits multiple craniofacial traits analogous to those of corythosaurian lambeosaurines, including the overgrown nasals forming a hollow supracranial crest, a steeply sloping frontal anterior platform, and an elevated ectocranial region of prefrontal. CT scans of the left nasal also indicate that *Q. changshengi* possesses a pair of novel accessory endonasal cavities with additional airflow pathways that would have functioned as breathing.

Resonant frequency calculations with comparisons among hadrosauroids further reflect that the novel endonasal airflow pathways in *Qianjiangsaurus changshengi* could help to facilitate vocalizations of relatively low frequencies partially overlapping with those in adult late-branching lambeosaurines. Therefore, the low-frequency vocalization within Hadrosauroidea is not tied to the hollow cranial crest formed by the premaxillae and nasals in lambeosaurines, but was instead realized via the independent acquisition of alternative acoustic structures operating under comparable functional constraints. In sum, the series of analogous skull features and similar low-frequency acoustic capabilities of nasal cavities between *Q. changshengi* and corythosaurian lambeosaurines reveal a prime example of morphological and functional convergence among hadrosauroids, which would have been helpful in relatively safe and efficient acoustic communication over long distances facilitating gregarious behavior. This phenomenon occurring during the Late Cretaceous can be effectively explained by adaptive evolution under similar selection pressures and developmental constraints due to gene pleiotropy, based on numerous extant vertebrate exemplars. Such selection pressures may be derived from similar environmental conditions (e.g., temperature and climate) and biological factors (e.g., predators and competition) during the same time interval (~late Santonian to early Campanian).

## Data Availability

Supplementary Materials for this study include geographic, stratigraphic and taphonomic information on the skull, close-ups and measurements of skull details, raw CT images of the left nasal, analyzed and resulting data of phylogenetic evaluations, and descriptions on calculations and comparisons of resonant frequencies, and is available online. More phylogenetic and 3D data presented in the study are openly available in Zenodo at https://doi.org/10.5281/zenodo.18719453.

## Supplementary Materials

The following supporting information can be downloaded at: https://www.mdpi.com/article/10.3390/biology15080615/s1. Figure S1: Geographic and stratigraphic distribution of *Qianjiangsaurus changshengi* in southeast Chongqing Municipality, southwest China. Figure S2: Satellite map showing the specific location of the quarry of the skull CIP V0002. Figure S3: Original taphonomic condition of the partial skull CIP V0002 (except for the braincase) along the 10th layer of the Late Cretaceous Zhengyang Formation. Figures S4–S13: Close-ups of skull details. Figures S14–S17: Digitized slices of the left nasal in cross section. Figure S18: Strict consensus tree recovered from the phylogenetic analysis of Hadrosauroidea, with the corresponding number for each node. Figure S19: Skulls of selected non-lambeosaurine hadrosauroids showing the airflow pathway through the nasal cavity proper. Figure S20: Skulls of selected lambeosaurines showing the airflow pathways through the nasal cavity proper and lateral diverticulum. Figure S21: Boxplots showing statistical comparisons of resonant frequency ranges among non-hadrosaurid hadrosauroids, lambeosaurines and saurolophines at the levels of the 1st to 5th harmonics using analysis of variance (ANOVA). Table S1: Linear measurements (in mm) of selected skull elements or structures of *Q. changshengi* based on the holotype CLGRP V00016 and referred specimen CIP V0002. Table S2: Estimated resonant frequencies on the primary airway of the nasal cavity among hadrosauroids, with comparisons of the equivalents on the addition airflow pathways via the anterodorsal and anteroventral passages. Table S3: Estimated resonant frequencies on the additional airway of the nasal cavity through the lateral diverticulum among lambeosaurines. Tables S4 and S5: Results of pairwise *t*-tests comparing resonant frequency ranges of five resonance modes (the 1st to 5th harmonics) among non-hadrosaurid hadrosauroids, lambeosaurines and saurolophines. Refs. [68,69,70,71,72,73,74,75,76,77,78,79,80,81,82,83,84,85,86,87,88,89,90] are cited in the Supplementary Materials.
